# Spontaneous Closure of a Coronary Artery Bypass Graft Pseudoaneurysm Embedded in a Mediastinal Hematoma

**DOI:** 10.1155/2023/9335392

**Published:** 2023-03-06

**Authors:** Daniel L. Hess, Gaither W. Horde, Karan Sarode, William S. Morgan, Salmaan Z. Kamal, Jubal R. Watts, Gregory D. Chapman

**Affiliations:** ^1^Department of Medicine, University of Alabama at Birmingham, Birmingham, AL, USA; ^2^Division of Cardiology, Department of Internal Medicine, Texas Tech University Health Sciences Center El Paso, El Paso, TX, USA; ^3^Department of Radiology, University of Alabama at Birmingham, Birmingham, AL, USA; ^4^Division of Cardiology, Department of Medicine, University of Alabama at Birmingham, Birmingham, AL, USA

## Abstract

Coronary artery bypass graft (CABG) pseudoaneurysms are a rare but often unrecognized clinical entity. They are prone to rupture and hemodynamic compromise and should therefore be on the differential in the appropriate patient. We present a case of a gentleman with a recent CABG surgery who presented with acute onset dyspnea and a large pleural effusion. Imaging revealed a saphenous vein graft pseudoaneurysm embedded in a mediastinal hematoma. Four weeks later, prior to planned stenting, the pseudoaneurysm had spontaneously closed. This case highlights an unusual acute presentation of a CABG pseudoaneurysm and a multidisciplinary approach to its management.

## 1. Introduction

Pseudoaneurysms of coronary artery bypass graft (CABG) vessels are rare, estimated to develop in <0.1% of grafts [1]. Pseudoaneurysms, or false aneurysms, are disruptions of the vessel wall that remain bounded by the outermost adventitial layer, with formation of a hematoma at the site of disruption. This is in contrast with true aneurysms, which are disruptions of all three layers of the vessel wall (intima, media, and adventitia). Because they lack the smooth muscle cell-rich medial layer that provides vascular wall integrity, pseudoaneurysms are more prone to rupture than aneurysms [2]. Because of their rarity, there is no consensus on optimal treatment modality of CABG pseudoaneurysms. Here, we present a case of a gentleman who presented with acute onset dyspnea, who was found to have a CABG pseudoaneurysm leading to a mediastinal hematoma and hemothorax.

## 2. Case Presentation

**Figure 1:**
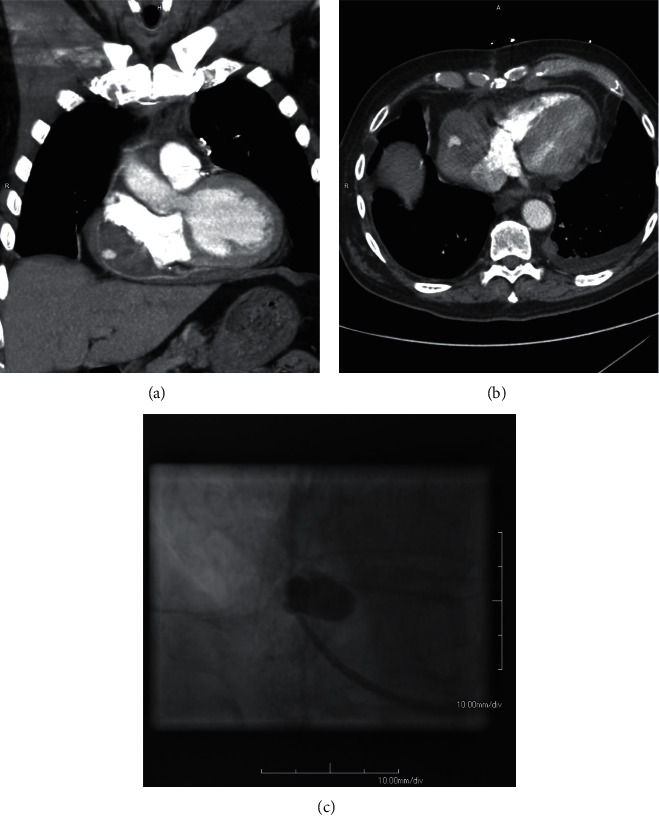
Pseudoaneurysm arising from a CABG graft. (a) and (b) A blush of contrast consistent with a potential pseudoaneurysm could be seen arising from the SV aorta-rPDA graft. It was embedded in a mediastinal hematoma. (c) Left heart catheterization confirmed the presence of a pseudoaneurysm arising from the SV aorta-rPDA graft.

**Figure 2:**
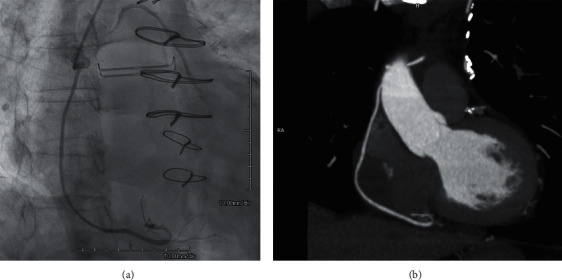
Spontaneous closure of pseudoaneurysm. (a) During left heart catheterization approximately four weeks after initial presentation, there was no detectable pseudoaneurysm, consistent with spontaneous closure. (b) On follow-up CT imaging approximately three and a half months after initial presentation, there was again no detectable pseudoaneurysm and the mediastinal hematoma had decreased in size.

A 73-year-old male with a medical history of heart failure with reduced ejection fraction (40%), diabetes mellitus, atrial fibrillation (on apixaban), chronic obstructive pulmonary disease, and three vessel CABG [saphenous vein (SV) aorta-right posterior descending artery (rPDA), SV aorta-obtuse marginal, and left internal mammary artery-proximal left anterior descending] approximately six months prior to presentation, presented with the acute onset of dyspnea while walking up a ramp at his home. The dyspnea continued for two days until his presentation to the emergency department. On admission physical examination, he had increased work of breathing and decreased breath sounds over the left lung base. Labs were significant for anemia (hemoglobin 8.5 g/dL) and undetectable serial troponins. Initial chest X-ray showed bibasilar opacities. Subsequent computed tomography (CT) chest without contrast showed a soft tissue density within the anterior mediastinum and pericardial cavity as well as a loculated left-sided pleural effusion. A thoracostomy tube was inserted, and pleural studies were consistent with an exudative effusion with over 2.5 million red blood cells but no malignant cells or infectious process. The chest tube output totaled approximately 3 L of bloody fluid over the next 5 days before the output stopped, and the chest tube was able to be removed. CT chest with contrast was then obtained, which showed a 1.5 cm blush of contrast at the level of the SV aorta-rPDA graft that was embedded in the same mediastinal mass seen on prior CT (Figures 1(a) and 1(b)). This suggested a pseudoaneurysm arising from the CABG graft that was embedded within a mediastinal hematoma. Subsequent left heart catheterization confirmed a pseudoaneurysm arising from the SV aorta-rPDA graft measuring approximately 1.5 cm in diameter (Figure 1(c); Video 1). This led to consultation with our cardiology and cardiothoracic surgery colleagues. The most likely explanation for the patient's presentation was a disruption in the SV graft pseudoaneurysm leading to a mediastinal hematoma and hemothorax. The patient was not a good surgical candidate given relatively recent CABG and his multiple medical co-morbidities. Ultimately, we decided to proceed with placement of an off-label covered stent across the site of the pseudoaneurysm. However, placement of an off-label covered stent required Institutional Review Board approval for emergent use. Although there was concern the pseudoaneurysm could further expand, the halted output from the chest tube as well as the patient's hemodynamic stability suggested that the pseudoaneurysm was contained. Because of this and the time required for approval of the stent, the patient was discharged with strict return precautions with plan for direct admission in three weeks for repeat left heart catheterization and placement of a covered stent across the pseudoaneurysm. The only medication change made on discharge was continuing to hold his apixaban for his atrial fibrillation given the risk of further expansion of his mediastinal hematoma. Repeat left heart catheterization three weeks after discharge showed a patent SV aorta-rPDA with spontaneous resolution of the pseudoaneurysm (Figure 2(a); Video 2), thus precluding use of the stent. Intravascular ultrasound confirmed closure of the pseudoaneurysm, although a minimal remnant pseudoaneurysm could be seen. The patient was doing well at cardiology follow-up approximately two months later. Cardiac CT with three-dimensional angiography approximately three and a half months after initial presentation showed persistent resolution of the pseudoaneurysm, with a hematoma that had decreased in both size and density (Figure 2(b)).

## 3. Discussion

Treatment approaches for CABG pseudoaneurysms include open surgical repair, endovascular interventions such as placement of covered stents, or observation [1]. There is no consensus on optimal treatment because there is limited data and no randomized controlled trials comparing one approach versus another. Previous literature suggests that they should be considered for repair if large (e.g. >1 cm) or if associated with symptoms [3]. In our case, we concluded that the patient's presenting symptoms were related to the pseudoaneurysm, after ruling out other potential etiologies. Since the pseudoaneurysm was embedded in a mediastinal hematoma, there was an initial disruption in the wall of the pseudoaneurysm, with hemostasis later achieved by tamponade from accumulating hematoma. When the SV was harvested during surgery, clips were placed on the branches to promote hemostasis. The most likely cause of the bleed was a dislodgement of a clip on one of the SV branches, leading to blood loss from the midportion of the graft. While it seems most likely that dislodgement of a clip led to the bleed, the patient was also on apixaban chronically for atrial fibrillation. We cannot rule out that apixaban contributed to expansion of the bleed, though apixaban was held throughout the hospitalization and on discharge. Additionally, because the epicardial layer is stripped during bypass graft operations, it is possible a communicating tract formed between the mediastinum and pleural space, leading to the patient's hemothorax. Indeed, there have been descriptions of CABG pseudoaneurysms causing complications including expanding to the point of compression of the right atria, ventricle, or main pulmonary artery [1, 4]. Another report describes a patient with chest pain who went for cardiac catheterization which showed a pseudoaneurysm with active extravasation. The patient died of cardiogenic shock before procedural intervention could be performed [5]. In our case, given the pseudoaneurysm was embedded in hematoma, indicative of prior bleed, it seemed most appropriate to intervene, especially given its size and risk of potential expansion and/or rupture. Successful closure of a SV graft pseudoaneurysm using a covered stent has previously been reported [6]. In our case, it was fortunate the pseudoaneurysm spontaneously closed, but there were no indications or data to suggest it would. The rate of spontaneous closure of CABG pseudoaneurysms is unknown, both because of their rarity and because many likely go undetected given lack of symptoms. However, to our knowledge, this is only the second report of the spontaneous closure of a CABG pseudoaneurysm [7]. In the end, diagnosis and management of this rare clinical entity required a multidisciplinary approach and ultimately resulted in an equally rare outcome.

## Data Availability

Original figures and videos related to the case can be made available after publication, upon request.

## Abbreviations

CABG: Coronary artery bypass graft
SV: Saphenous vein
PDA: Posterior descending artery.
